# Patches as Polymeric Systems for Improved Delivery of Topical Corticosteroids: Advances and Future Perspectives

**DOI:** 10.3390/ijms232112980

**Published:** 2022-10-26

**Authors:** Natallia V. Dubashynskaya, Yury A. Skorik

**Affiliations:** Institute of Macromolecular Compounds of the Russian Academy of Sciences, Bolshoy pr. V.O. 31, 199004 St. Petersburg, Russia

**Keywords:** patches, topical corticosteroids, oromucosal drug delivery

## Abstract

Mucoadhesive polymer patches are a promising alternative for prolonged and controlled delivery of topical corticosteroids (CS) to improve their biopharmaceutical properties (mainly increasing local bioavailability and reducing systemic toxicity). The main biopharmaceutical advantages of patches compared to traditional oral dosage forms are their excellent bioadhesive properties and their increased drug residence time, modified and unidirectional drug release, improved local bioavailability and safety profile, additional pain receptor protection, and patient friendliness. This review describes the main approaches that can be used for the pharmaceutical R&D of oromucosal patches with improved physicochemical, mechanical, and pharmacological properties. The review mainly focuses on ways to increase the bioadhesion of oromucosal patches and to modify drug release, as well as ways to improve local bioavailability and safety by developing unidirectional -release poly-layer patches. Various techniques for obtaining patches and their influence on the structure and properties of the resulting dosage forms are also presented.

## 1. Introduction

Topical corticosteroids (CS) are the drugs of choice for the symptomatic treatment of various diseases of the skin (e.g., atopic dermatitis and psoriasis) [1,2] and the oral mucosa (e.g., oral lichen planus, aphthous stomatitis, pemphigus, etc.) [3,4,5,6]. Their high clinical efficacy is associated with their anti-inflammatory, immunosuppressive, antiproliferative, and vasoconstrictive effects [7,8].

High-potency topical CS (e.g., clobetasol propionate and betamethasone dipropionate) is clearly safer and more effective than oral dosage forms; however, severe side effects can occur with their long-term use [9,10,11].

Pharmacokinetic and pharmacodynamic parameters indicate that the development of modified dosage forms for topical administration of CS (e.g., mucoadhesive polymeric matrices for mucosa and skin applications) is of primary relevance. The use of different types of polymeric carriers for this type of administration can increase the drug residence time at the target site due to bioadhesion while also ensuring controlled and prolonged release. In general, a perfect CS carrier would provide a safe and effective dosage through targeted delivery while also reducing the drug dose, the frequency of administration, and any side effects by increasing the local bioavailability [12,13,14].

Although topical administration of CS is generally considered safe, the systemic absorption of these drugs can cause severe side effects; therefore, new delivery systems are being actively researched [15,16]. Various new drug delivery systems (e.g., solid nanoparticles, polymer polyelectrolyte complexes, and nanostructured lipid particles) are being used to improve local bioavailability by increasing transmucosal and transdermal CS absorption. The use of these systems increases drug accumulation in the target tissue and improves the risk/benefit ratio by reducing side effects [17,18,19,20]. Nevertheless, these drug carriers do not yet provide the same reliable protection against undesirable systemic absorption and associated systemic toxicity that is presently achieved with systems providing unidirectional release (e.g., poly-layer patches with drug-impervious protective layers) [21,22].

Previously, many articles have discussed the general development of polymeric mucoadhesive systems for local drug delivery (including CS delivery) [23,24,25,26] or have examined specific properties of these systems, such as bioadhesion or the composition of mucoadhesive polymers [27,28]. However, only a limited number of studies in the literature have described the use of bioadhesive patches as polymeric oromucosal drug delivery systems to improve the local bioavailability and local therapeutic effects of CS. In this review, we have focused on the biopharmaceutical background, now leading to the development of effective and safe mucoadhesive patches for topical CS delivery, and we have discussed the most important physicochemical characteristics of these polymeric carriers.

## 2. Patches as Oromucosal Drug Delivery Systems for Topical Application

**Figure 1:**
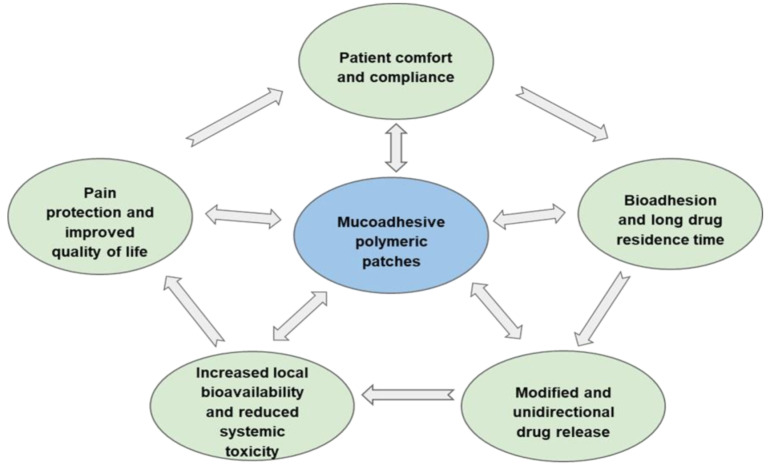
The main biopharmaceutical advantages of patches as optimal dosage forms for oromucosal delivery of topical corticosteroids.

Patches are most often polymeric films or electrospun fiber-based materials (in the literature, the terms “film” and “patch” are often used interchangeably [29]); they are relevant new and patient-friendly dosage forms for topical delivery of various drugs, including CS [30,31,32]. The usability of mucoadhesive polymeric patches for application to the mucosa improves patient compliance compared to traditional liquid (mouthwashes and spray) and soft (gels, ointments and pastes) dosage forms [23,31]. Furthermore, the traditional dosage forms have low local drug bioavailability in the oral mucosa due to the short residence time, as well as a high risk of systemic toxicity due to the dissolution of the drug in saliva, ingestion into the gastrointestinal tract, and subsequent absorption into the systemic circulation [4]. By contrast, due to bioadhesion, the patch adheres securely to the oral mucosa, and the drug release can be targeted to the oral cavity or the oral mucosa [29]. Patches that release the drug into the oral mucosa increase local bioavailability by blocking non-targeted drug absorption in the gastrointestinal tract, thereby significantly reducing drug dose, systemic toxicity, and frequency of side-effects [33]. Oral patches can also protect the damaged tissue from various factors affecting pain receptors to reduce pain and increase the effectiveness of treatment, while also improving the quality of life [4,29,34]. The patches appear to have all the necessary characteristics of an optimal dosage form for improved oromucosal delivery of topical CS (Figure 1).

### 2.1. Optimal Patch Requirements for Oromucosal Delivery of Topical Corticosteroids and Approaches to Achieving Them

In general, the advantages of patches correspond to the requirements for optimal systems with improved oromucosal delivery of CS (Table 1). To be safe and effective, polymeric patches for CS delivery with enhanced local bioavailability should have the following characteristics: (i) prolonged mucoadhesion to the oral mucosa and the injured site; (ii) controlled and modified release of the active pharmaceutical ingredient; (iii) targeted drug release and distribution in the mucosa; (iv) low systemic absorption; (v) biodegradability and dissolution of the polymeric platform during drug release; (vi) safety, nontoxicity, and biocompatibility of polymer composition components; and (vii) maximum patient compliance and comfort [4,34].

Therefore, the pharmaceutical R&D of these new dosage forms should consider the influence of important biopharmaceutical factors on drug efficacy. 

Foremost is the physical state of the drug substance; for instance, compounds in the amorphous state have better solubility and increased dissolution rates compared to their crystalline counterpart, so they have biopharmaceutical advantages for drug delivery [31,35]. The second is the nature of the excipient (polymer base, plasticizers, and solubilizers), as this is greatly influenced by the quality of the patches (mucoadhesion, as well as cross-linking density and flexibility). For example, the presence of chemical groups capable of forming hydrogen bonds (hydrogen bonding capacity) or protonated in a weakly alkaline environment (positive charge) and the molecular weight (MW) of polymers (usually above 100,000) can ensure strong bioadhesion to the oral mucosa [27]. Moreover, the many viscosity classes and MWs of the polymers provide large variability in physicochemical properties and have formed the basis for successful applications in pharmaceutical R&D [36]. The third factor is the type of patch (single-layer or poly-layer patch), as this may have a significant impact on both bioavailability and toxicity. For example, the use of poly-layer patches allows both the programming of the drug release profile and directional diffusion of the drug diffuses toward the damaged site, thereby minimizing ingestion into the gastrointestinal tract [26]. Finally, the method of patch creation is of great importance for establishing the desired properties and characteristics. Various technological procedures, such as solvent evaporation [22], electrospinning [37,38], sublimation [39], compression with a hydraulic press [40], and 3D printing technology [41,42], create different patch forms, including polymer films or non-woven materials, hydrogels, and cryogels. In addition, patches can be produced using layer-by-layer technology to create a structure by consecutive deposition of polymers with opposite charges [43,44]. The matrices obtained by various methods differ in the most important physicochemical characteristics (swelling and porosity), thereby affecting the mucoadhesion of these systems and the rate of drug release. For example, the swelling of nanofibers is an important property for successful bioadhesion, and the degree of swelling depends significantly on the rapidity of hydration of the polymeric nanofibers and subsequent gelation on the moist surface of the mucosa [45]. The influence of biopharmaceutical factors on the efficacy of patches as oromucosal dosage forms is summarized in Table 2.

Optimal patches for oromucosal administration must also have specific technological characteristics:

(i) polymer matrix thicknesses of 50 μm to 1000 μm [46];
(ii) suitable mechanical properties (strength and elasticity) [47];
(iii) wettability and swelling as crucial properties for bioadhesion and drug release [45];
(iv) structural integrity during hydration [31];
(v) weakly acidic or weakly alkaline surface pH (5.5–8.2), as strongly acidic or strongly alkaline films cause mucosal irritation and discomfort, as well as cytotoxicity [31,48].

Various strategies are used for the R&D of oromucosal patches with the desired properties. Key approaches for creating patches with improved characteristics are discussed below.

### 2.2. Strategies for Increasing the Bioadhesion of Oromucosal Patches for Topical Application

Many nanotechnology-based drug delivery systems for topical oral administration are ineffective because the sticky and viscous mucus layer that protects the body from foreign particles and pathogens also acts as a physical barrier to delivery systems [49,50,51].

**Figure 2:**
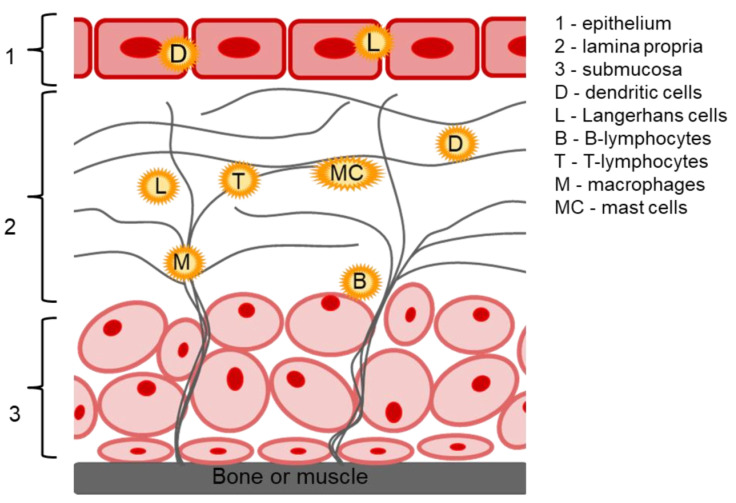
The structure of oral mucosa.

The oral mucosa has important protective and barrier functions; the main structural parts of the oral mucosa are the epithelium, the lamina propria and the submucosa (Figure 2). The oral epithelium is a stratified squamous epithelium containing multiple cell types in discrete layers; it may be both keratinized (epithelium of the hard palate and gingiva) and non-keratinized (epithelium of the sublingual and buccal mucosa) [52]. It is damage to the oral epithelium that leads to the development of various ulcers, including oral lichen planus [53]. The lamina propria is a connective tissue consisting of cells, blood vessels, nerve and collagen fibers, as well as immunocompetent cells such as macrophages, B- and T-lymphocytes, which are responsible for acute and chronic inflammation, and mast cell secreting inflammatory mediators and vasoactive agents (histamine, and heparin). In addition, oral mucosa contains clusters of immune system cells (Langerhans cells and dendritic cells), both of which may also mediate the development of autoimmune diseases of the oral mucosa [51]. The submucosa provides a flexible attachment function to underlying structures (bone or muscle) [54]. Local CS delivery to the site of inflammation allows the effective influence of all the damaging factors due to anti-inflammatory, immunosuppressive, and hypersensitizing action caused by genomic and non-genomic effects of CS (a detailed mechanism of CS action is described in our recent review [12].

The low permeability (barrier property) of the oral mucosa layers predominantly affects drug absorption into the systemic bloodstream (sublingual and transbuccal administration). For this review, a discussion of the structure and properties of the mucus-coated epithelium is important. The mucus is a hydrogel consisting mainly of 1–5% water-insoluble glycoproteins, 95–99% water, and minor amounts of other components, such as proteins, enzymes, electrolytes, nucleic acids, and macromolecules [27,49,50]. Mucus has a reticular structure formed by entangled glycosylated protein fibers called mucins. Mucins are mainly composed of glycans (40–80%), which, at physiological pH, are linked with negatively charged proline, threonine, and serine domains through sialic acid residues in the terminal end. The presence of sialic acids and sulfonic acids means that mucus is a polyanionic layer that is able to interact with polycations. In addition, mucus has hydrophobic cysteine-containing domains that form globular structures due to disulfide bonds [49,55]. 

One strategy for overcoming the physical barrier of the oral mucosa is to use mucoadhesive compounds [56,57]. Mucoadhesion of drug-containing polymeric carriers to mucus significantly increases the residence time of the drug on the mucosa, thereby providing sustained, prolonged, and localized drug release [49,50,58].

**Figure 3:**
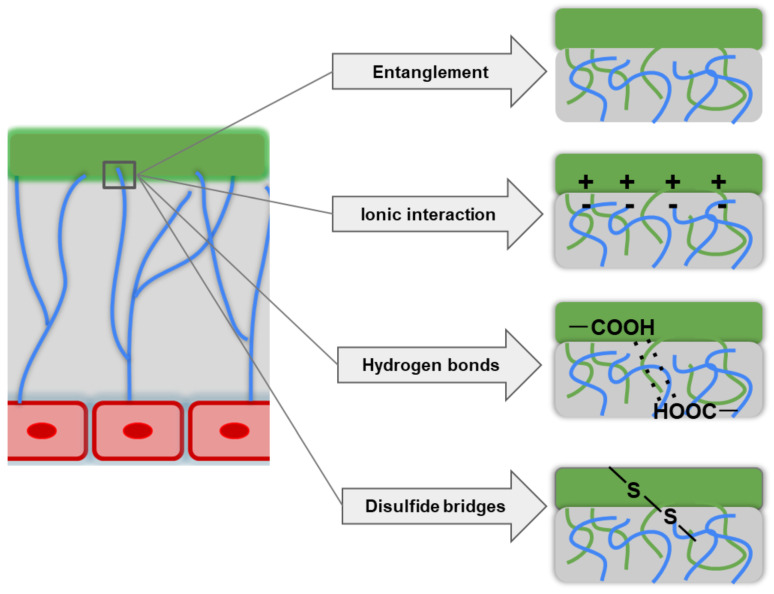
Illustration of various bioadhesion mechanisms.

In principle, bioadhesion is realized through different intermolecular interactions between the polymer and mucin (Figure 3). The mechanisms of bioadhesion are described in detail by the following theories: (i) adsorption, (ii) diffusion, (iii) electronic interaction, (iv) fracture, (v) mechanical interaction, and (vi) wetting [27,45,59].

The adsorption theory explains bioadhesion as being due to the formation of primary and secondary chemical bonds of covalent and non-covalent nature (hydrogen bonds and hydrophobic interactions between nonpolar radicals, electrostatic interactions, and van der Waals forces) during contact between the mucoadhesive polymer and the mucus. The diffusion theories are based on the entanglement of polymer chains with mucus glycoproteins to form an entangled network. The key characteristics of mucoadhesive polymers that affect diffusion properties are the flexibility of the polymer chain, the similarity of chemical structures, and the diffusion coefficient. The electronic theory focuses on the different electronic properties of polymers and the mucus glycoprotein, as these differences promote electron transfer between the two surfaces and the formation of a charged double layer in that area. The result is the formation of forces of attraction and interdiffusion interactions at the mucus/polymer interface. The fracture theory studies the strength of the polymer/mucus adhesive bond as a function of the force required to detach the polymer from the mucus. In this case, the strength of mucoadhesion increases with the lengthening of the mesh chains and the reduction of the degree of cross-linking. The mechanical theory suggests that adhesion results from the interlocking of the polymer with the rough mucus surface irregularities; the rough surface also provides increased surface area and enhanced viscoelastic and plastic energy dissipation during the failure of this connection. The wetting theory describes the ability of the polymer to spread over the epithelial surface (as a rule, moderately wetted polymers have optimal adhesion) [27,45,59]. 

Mucoadhesive polymers must have specific characteristics (Table 3), such as hydrogen bonding functional groups, a positive surface charge, optimal wettability, high viscosity, and a high degree of swelling (hydrogel-forming properties), as well as high flexibility of the polymer chain for binding and entanglement to the mucoadhesive reticulum [59]. 

The mucoadhesion of the polymer also depends on the polymer chain length (i.e., the optimal polymer chain length should be long enough to promote the interpenetration but short enough to facilitate diffusion). The degree of cross-linking is also important, as highly cross-linked polymers swell in water and retain their structure, thereby increasing the polymer/mucus interpenetration and providing controlled drug release. However, as the cross-linking increases, the chain mobility decreases and reduces mucoadhesiveness. The spatial conformation also comes into play because, unlike linear polymers, the helical conformation of dextrans shields adhesive groups [60,61]. As a rule, high molecular weight linear polymers containing many hydrophilic negatively charged functional groups and capable of forming 3D structures have excellent bioadhesive properties [27,50]. In addition, these polymers should have characteristics that include safety, non-toxicity, and biodegradability.

The main approaches for obtaining bioadhesive patches are the use of mucoadhesive polymers [61] and the modification of the polymer film surface with bioadhesive components [34].

## 3. Bioadhesive Polymers

Among bioadhesive polymers, natural polysaccharides, such as chitosan and its derivatives, sodium alginate (ALG) [39,62,63,64,65], and a number of synthetic polymers, such as polyethylene oxide (PEO), polyvinylpyrrolidone (PVP), polymethacrylate, polyacrylic acid (PAA) and poloxamers (pluronics, block co-polymers of polyoxyethylene and polyoxypropylene) [4,31,49,66,67]), have particular advantages for the R&D of oromucosal patches due to their high mucoadhesive properties. Cellulose derivatives, e.g., methyl cellulose, hydroxypropyl methyl cellulose (HPMC), and carboxymethyl cellulose (CMC), are widely used due to their excellent mucoadhesive and hydrogel-forming properties [37,68]. 

Prof. Khutoryanskiy classifies mucoadhesive polymers, such as chitosan, ALG, PAA, and cellulose, as first -generation (non-specific) mucoadhesive materials. Second -generation (specific) mucoadhesive materials include polymers modified by chemical conjugation of the polymers with molecules bearing thiol, catechol, boronate, acrylate, methacrylate, maleimide, and N-hydroxy(sulfo)succinimide ester groups to improve their mucoadhesive properties [59].

In the following, we review the main bioadhesive polymers that are most commonly used for the development of mucoadhesive oral patches. Among these polymers, natural polysaccharides are especially important. Polysaccharides are non-toxic and biocompatible “green” biopolymers that are widely used in the development of patches for medical applications [69].

### 3.1. Cationic Mucoadhesive Polymers

Chitosan is a natural polymer that is widely used for various biomedical applications [70,71,72,73], including mucoadhesive drug delivery systems [74,75]. Chitosan, which consists of glucosamine and N-acetylglucosamine linkages, is obtained by the deacetylation of chitin and has a degree of deacetylation (DDA) typically varying from 50 to 95% [76]. In addition to the MW, the DDA is an important characteristic of chitosan since it characterizes the presence and number of free amino groups, which determine the different activities of this polymer [74,77,78]. The strong bioadhesion of chitosan is explained by the presence of positive charges due to the protonation of amino groups under physiological conditions; thus, chitosan shows better mucoadhesion compared to anionic polymers due to its capacity for electrostatic interactions with the negatively charged mucosal surface [79,80]. The cationic nature further determines the antimicrobial and antifungal activity of chitosan [81,82,83]. Chitosan also has the ability to swell in an aqueous medium by relaxation of chains due to the solvation of hydrophilic groups, and the hydrogel formed by swelling is an important factor influencing drug release patterns [48]. Thus, swelling is an important property of chitosan membranes, especially those for oral use [84]. The addition of hydrophobic compounds reduces the swelling of polysaccharide membranes [85], whereas various hydrophilic compounds such as hydroxypropyl β-cyclodextrin (HPβCD) and triethanolamine improve the solvation of polymer chains and increase swelling [62,86]. 

Chitosan has known wound-healing and anti-ulcer activity and displays synergistic effects with anti-inflammatory drugs, especially with CS [12,14,75,87]. However, most CS are hydrophobic; therefore, their introduction into hydrophilic polymer matrices requires special approaches to achieve uniform distribution (e.g., the use of various nanocontainers, including complexes with cyclodextrin (CD) and its derivatives, and the addition of co-solvents) [88,89].

Chitosan has been used successfully in the pharmaceutical R&D of oromucosal patches for topical application. For example, do Nascimento et al. [62] used medium MW chitosan (viscosity 200–800 cP and DDA of 75–85%) to create bioadhesive triamcinolone acetonide (TA) films for topical application in the oral cavity. The uniform dispersion in chitosan membranes was ensured by including TA in complexes with βCD or HPβCD and adding triethanolamine as a co-solvent. The resulting patches had optimal mechanical properties (tensile strength of 70–90 N and elongation at break of 10–20%), as well as a weakly acidic surface pH (5.5–6.0) and a thickness of 40–65 μm. The chitosan matrices with HPβCD and triethanolamine had the most uniform drug content. The addition of both CDs (βCD and HPβCD) and triethanolamine significantly improved the swelling rate (1.5-fold) compared to the original chitosan film containing TA only. At the same time, the presence of CDs and triethanolamine prolonged TA release by approximately 2-fold (40–50% of the drug was released within 24 h from the modified films versus 80% from the original chitosan films). 

Esfahani et al. [63] obtained clobetasol-containing chitosan (MW 190,000–310,000; DDA 75–85%) patches by electrophoretic deposition for use in the local treatment of oral diseases. The obtained systems had a thickness of 80 μm and showed swelling of 200–360% and good mechanical properties (Young’s modulus of 0.6 MPa and stress at break of 0.55 MPa); moreover, about 80% of the clobetasol was released in 2 h.

### 3.2. Anionic and Non-Ionic Mucoadhesive Polymers

The mucoadhesion mechanisms of anionic and non-ionic polymers have been widely discussed. Despite their incompatible charges, anionic polymers are capable of mucoadhesion primarily due to their side carboxyl groups, especially in optimal pH ranges [59,90].

ALG is a mucoadhesive natural polysaccharide consisting of mannuronic acid and guluronic acid organized in homogeneous or heterogeneous blocks. ALG is capable of green chemical cross-linking with divalent metals and has a high water-absorption capacity, as well as desirable viscoelastic and mechanical properties [91,92]. ALG exhibits good mucoadhesive properties due to the presence of free carboxyl groups that can interact with mucin by hydrogen bonding. Soluble ALG also produces a viscous and cohesive 3D hydrogel structure that enhances mucoadhesive interactions [93]. High -viscosity polymers, such as ALG, have good mucoadhesive properties because, according to the diffusion theory of bioadhesion, both high viscosity and high MW ensure that the polymer chains penetrate the mucosa to a depth sufficient to create strong adhesive bonds by entanglement [94]. Moreover, due to their high porosity, ALG-based polymer patches also effectively load various drugs [95]. For example, Okeke et al. [39] obtained a mucoadhesive buccal nicotine patch based on ALG and HPMC by solvent evaporation and sublimation methods. In contrast to the solvent-cast patches, freeze-dried patches have high porosity (60–75%), depending on the ALG content. The swelling profile of the developed patches also depended on the receipt method, as solvent-cast films showed a gradual increase in the swelling index within 30 min, while the swelling index of freeze-dried patches increased rapidly for a short time (2 min) and then remained constant. The mucoadhesion values were also higher for patches obtained by solvent evaporation than by freeze-drying, and the mucoadhesion properties increased for both systems with increasing ALG content. The rate of drug release was highly dependent on the ALG amount, as patches with the highest ALG concentration released 90% and 100% of nicotine within the first 30 min and 4 h, respectively, while patches containing minimal ALG addition provided prolonged release (less than 60% of nicotine within 4 h).

#### 3.2.1. Cellulose Derivatives

Cellulose derivatives, such as anionic sodium CMC and non-ionic HPMC, hydroxyethyl cellulose (HEC), and ethyl cellulose, are suitable biopolymers for oral patches and show good mucoadhesiveness, swelling, and physicochemical properties [37,96,97,98]. 

CMC, a polyanionic polymer, has stronger bioadhesive properties compared to most non-ionic cellulose derivatives because its hydrophilic -CH_2_COOH groups affect both water absorption and hydrogen bonding [96]. Ramineni et al. [68,99] developed bi-layer patches consisting of PVP and CMC for the topical treatment of oral dysplasia. Poly (ethylene-co-vinyl acetate) was used as the backing layer. The developed patches had a thickness of 0.30–0.39 mm and were characterized by excellent bioadhesive properties; the ex vivo mucoadhesion time was 6–10 h. Laffleur et al. [37] developed promising drug formulations for the treatment of various oral diseases in the form of bioadhesive patches based on ethyl cellulose with the addition of HPMC and HEC and their mixtures with PVP or PEO as a plasticizer. The obtained polymer films had a thickness of 90–200 nm and a surface pH of 6 and varied in their values of folding endurance depending on the composition. The addition of HPMC to the formulation increased the folding endurance (over 300-fold) compared to non-HPMC compositions (40-fold). The developed patches showed excellent bioadhesion to porcine buccal mucosa; with ethyl cellulose/HEC or ethyl cellulose/HPMC-based formulations achieving the longest adhesion time (3–6 h).

#### 3.2.2. PAA and PAA Derivatives

The commercially available synthetic polymer PAA and its derivatives are characterized by high mucoadhesive properties [100]. Various polymers, such as polycarbophil, several types of carbomer homopolymers and copolymers, have been confirmed as successful mucoadhesive matrices for the development of oral patches. Chemically, these polyacrylates are either linear PAA chains or high MW PAA derivatives that have been modified by crosslinking with divinyl glycol, allyl pentaerythritol, or allyl sucrose [101].

The bioadhesion of PAA arises mainly due to its hydrophilic nature, which promotes the wettability of the polymer and ensures strong adhesive contact with the mucosa [94,102]. The MW and cross-linking density of PAA affect its mobility and flexibility by reducing the effective length of the polymer chain that can penetrate and entangle within the mucosa and form adhesive bonds through this physical or mechanical interaction. However, the mucoadhesive properties of polyacrylates can vary significantly, depending on the experimental conditions and type of mucin [103,104,105]; therefore, their specificity should be evaluated when designing patches with specifically desired functional characteristics [101]. Chinwala et al. [94] developed oral patches for the delivery of thyrotropin-releasing hormone (TRH) based on different polymers, such as PAA, polycarbophil, Carbopol 934P, Carbopol 974P, and Carbopol 971P (MW of 700,000 to 3–4 billion), HPMC (MM of 1,200,000–1,800,000) and ALG, as well as various combinations of these polymers. The obtained patches had different mucoadhesive properties: the PAA- and HPMC-based patches exhibited maximum and minimum mucoadhesion, respectively; whereas ALG patches had adhesive characteristics intermediate between PAA and HPMC. Mixtures of PAA and either HPMC or ALG did not improve the mucoadhesive properties, as the mucoadhesion of the polymer combinations was mainly the result of the combination of adhesive strength, determined by the ratio of each polymer. The PAA, ALG, and HPMC patches had the highest, intermediate, and lowest hydration and swelling, respectively. These results correlate well with the bioadhesion data, confirming that swelling is the best indicator of high mucoadhesive properties (in turn, swelling occurs only with good polymer hydration). The type of polymer and the polymer mixture composition also influenced the loading efficiency of TRH: the maximum and minimum values of loading efficiency were found in ALG and PAA patches, respectively. At the same time, among the combinations of polymers, patches based on a mixture of PAA and ALG had the best TRH loading efficiency. An in vitro release kinetics study showed that only patches based on PAA provided sustained release of TRH (30% and 100% of the TRH was released in 0.5 and 8 h, respectively). By contrast, patches based on both ALG and HPMC released 80–100% of the TRH in 0.5 h. Combinations of PAA with either ALG or HPMC also provided a sustained TRH release (40% and 100% of the TRH was released in 0.5 and 4–6 h, respectively).

#### 3.2.3. Thiolated Mucoadhesive Polymers

The chemical interactions between mucoadhesive polymers and mucus are usually noncovalent (mainly hydrogen bonding and hydrophobic and electrostatic interactions). The thiol function is known to form covalent disulfide bonds with cysteine-rich mucus domains [49,106,107]. Therefore, the use of thiolated polymers (modified polymers that contain a thiol group) capable of forming covalent bonds with mucus components enhances the specificity of the interaction and improves the bioadhesive properties [27,108]. Thiolated polymers (such as PAA, CMC, starch, hyaluronic acid, pectin, and chitosan) can show significantly prolonged (up to 25-fold) mucoadhesion compared to the corresponding unmodified polymers, and a clear correlation exists between the number of thiol groups and mucoadhesion [47,109,110,111,112]. Duggan et al. [113] have synthesized thiolated derivatives of PAA (MW 450,000) and polyallylamine (MW 15,000) with the resulting thiol contents in the modified polymers of 400–487 μmol/g. Thiol modification of both polymers improved their swelling, cohesive, and mucoadhesive properties compared to unmodified control samples (the adhesion time of thiolated polymers increased up to 60 -fold).

Therefore, thiolated polymers with improved bioadhesion represent promising materials for creating patches for local oral applications. Ozkahraman et al. [114] produced oral patches based on modified κ-carrageenan and pectin-containing triamcinolone acetonide. κ-Carrageenan-g-acrylic acid was first modified with different thiolating agents (cysteine and 3-mercaptopropionic acid), and mixtures of the obtained κ-carrageenan derivatives and pectin at different ratios were used to prepare hydrogel patches by solvent casting methods. In this case, increasing the proportion of thiolated polymers in the resulting systems from 50 to 90% increased both the swelling (approximately two-fold) and the mucoadhesive properties (the force of bioadhesion increased by 1.1-fold and 1.3 -fold for formulations containing polymers functionalized with cysteine- and 3-mercaptopropionic acid, respectively). The developed modified patches also provided prolonged drug release for 7–8 h. Naz et al. [115] created a thiolated film for oral delivery of fluconazole based on chitosan modified with thioglycolic acid (170 μmol/g of thiol groups) and cysteine-modified CMC (380 μmol/g). The thiolated films obtained with an average thickness of 0.08 mm and a pH of 6 had an approximately two-fold higher water absorption capacity and an approximately six-fold higher mucoadhesion compared to unmodified films; in addition, controlled drug release from the film was achieved within 8 h. Hanif et al. [116] developed mucoadhesive oral patches based on arabinoxylan modified with thioglycolic acid by a solvent casting technique. The resulting films, which had a thickness of 0.150 mm and a surface pH of pH 6.6, contained 2800 µmol of thiol groups per gram of polymer. The films had an acceptable mechanical strength and mucoadhesiveness, with a folding endurance of over 300 and a force of bioadhesion of about 11 N. In addition, the developed polymeric compositions provided a prolonged release of the drug (85% of tizanidine was released over 8 h).

## 4. Bioadhesion Modifications of Surfaces

Surface functionalization using molecular design is a fundamental approach for imparting new functional properties to biomaterials [34,117]. Adhesion proteins (e.g., polydopamine) containing significant amounts of catechol (3,4-dihydroxy-L-phenylalanine) and amine (lysine) groups may be used for bioadhesion functionalization of polymer patch surfaces [34,118,119,120,121,122].

For example, Owji et al. [34] used polydopamine for bioadhesion modification of the surface of a drug delivery matrix based on polyhydroxyalkanoates (PHAs) targeted to the oral mucosa. PHAs are biodegradable, biocompatible, and elastomeric bacterially synthesized polymers with highly specific mechanical properties and low melting points [123,124,125,126]. Functionalizing the surface of PHA-based materials with a polydopamine coating can improve many properties, such as bioadhesion and hydrophobicity. The developed films showed improved hydrophilicity and bioadhesion and supported increased cell proliferation in vitro and neovascularization in vivo. 

## 5. Strategies for Drug Introduction into Polymer Patches and Modification of Drug Release 

Providing the correct characteristics and rate of drug release is an important challenge when developing effective oromucosal CS delivery systems. In general, loading the drug into hydrophilic polymeric matrices promotes a high drug release rate, especially for hydrophilic drugs [4,127], while the addition of oligosaccharides, such as CDs, prolongs drug release from films [62,128,129]. For example, d’Angelo et al. [4] developed films based on HPβCD and PEO for the local delivery of dexamethasone phosphate (DexP), a hydrophilic drug. The interaction between PEO and CD via hydrogen bonding was confirmed as the main factor ensuring excellent thermal and mechanical (tensile strength and elasticity) properties, as well as wettability of films, mucoadhesion to the oral mucosa, a suitable dissolution rate of the polymer composition, and a modified drug release profile [130]. The formation of host/guest complexes between CD and DexP [130,131] within the polymeric matrix also provided high drug loading (92%), homogeneous distribution throughout the film, and sustained release (45% and 100% of total DexP was released in 5 min and 15 min, respectively, into phosphate-buffered saline (PBS, pH 6.8); these amounts correlated with the dissolution rate of CD in the polymeric film).

The integration of hydrophobic drugs in hydrophilic hydrogel membranes is also a challenging task and requires special techniques to ensure homogeneous dispersion [132]. This problem can be overcome in several ways: (i) introducing hydrophobic ingredients into different nanocontainers (nanoparticles or liposomes) and then doping them in hydrophilic matrices [133,134,135]. (However, this multi-step approach involves additional development and characterization of CS nanocarriers, which increases the difficulty of the patch-obtaining process); (ii) forming CS inclusion complexes based on CD and CD derivatives (CDs act as both solubilizers of hydrophobic substances and prolongers of drug release [136]); (iii) dissolving the materials in suitable solvents, such as linoleic acid and acetate buffer; and (iv) applying co-solvents to solubilize water-insoluble compounds to achieve homogeneous dispersion of CS using a simple one-step procedure [4,62]; and (v) using sonication to intensify the dissolution process [99]. For example, Jug et al. [136] developed a mucoadhesive buccal patch with triclosan based on pectin and carbopol. The solubility of triclosan was optimized using βCD and β-cyclodextrin-epichlorohydrin (EPIβCD) and the anionic carboxymethylated EPIβCD (CMEPIβCD). The use of βCD resulted in a biphasic triclosan release profile dependent on the degree of hydration of the matrix, whereas EPIβCD and CMEPIβCD provided a constant release rate (a zero-order release kinetic) due to their high solubilizing effects.

The introduction of low MW active pharmaceutical ingredients disrupts the interaction between macromolecular chains; thus, the mechanical strength and elasticity of polymer patches decrease. Therefore, the elasticity of polymer matrices is regulated by adding various plasticizers (e.g., glycerin) [137,138]. The importance of drug interactions in the polymer network of membranes should also be considered when designing pharmaceutical patches; for example, CD affects the diffusion behavior of drugs within polymer films by changing the cell size of the polymer network. Consequently, CD can modify GC release [62,139,140]. The different approaches to modifying drug release from polymer patches are summarized in Table 4.

## 6. Methods to Improve the Local Bioavailability and Safety of Oromucosal Patches for Topical Application

**Figure 4:**
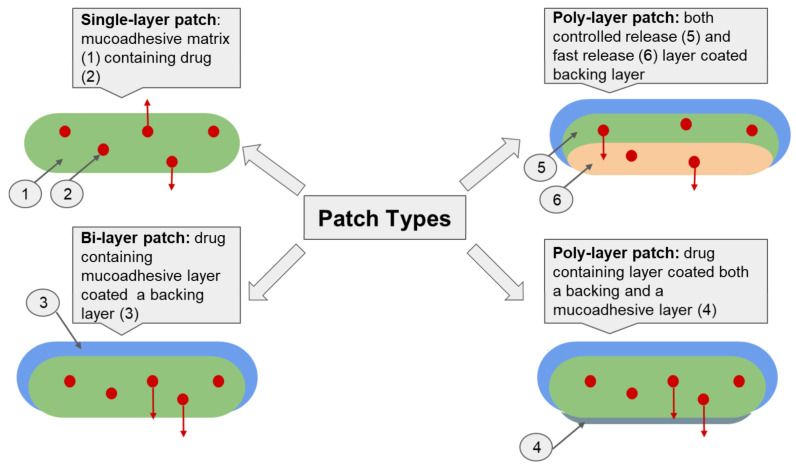
Illustration of various patch types.

The application of innovative dosage forms is an important biopharmaceutical aspect for providing highly effective therapy for oral mucosal diseases. In addition to single-layer patches, bi- and poly-layer patches are used to improve the biopharmaceutical properties of drugs, as they allow programmed drug release and enable unidirectional drug release (Figure 4). Targeted drug release into the mucosa prevents CS entry into the saliva or gastrointestinal tract and further systemic absorption. Consequently, both local bioavailability and therapeutic efficacy are improved, while systemic toxicity and the frequency of side effects are reduced. This strategy can be realized using poly-layer patches with an outer impermeable membrane that inhibits drug release [22,26,141]. 

Among the multilayer patches, we can identify the following types: (i) the bi-layer patch and (ii) the poly-layer (sandwich) patch (Figure 3) [31,47]. Bi-layer patches usually consist of an impermeable backing (protective) layer and a drug-containing bioadhesive layer [142]. Hydrophobic polymers, such as polycaprolactone (PCL) and ethyl cellulose, are used to fabricate the protective layer [31,91,143]. Poly-layer patches usually have three or more different layers: (i) backing/controlled drug release/fast drug release layers; (ii) backing/drug containing/mucoadhesive layers [47].

### 6.1. Electrospinning Technology

Electrospinning is a universal method for producing both single- and poly-layer patches. Electrospinning provides the possibility of combining polymers with different properties and drug molecules, thereby simulating the optimal physical structure and effective biomedical functionality of the created materials. Therefore, electrospinning patches have high porosity and surface area, which improve both the drug bioavailability and the level of adhesion to the oral mucosal epithelium. In addition, the sequential layering of polymer fibers with different physicochemical characteristics (mainly solubility) is a simple and convenient technique for obtaining multilayer matrices [38,47,144]. For example, Colley et al. [31] obtained a clobetasol-containing bilayer mucoadhesive patch by electrostrospinning. The developed patch (an average thickness of 400 µm; surface pH of 8.0) consisted of an outer hydrophobic layer of PCL (MW 80,000) and an inner mucoadhesive layer of PVP (MW 2,000,000) and Eudragit^®^ RS100 (a copolymer of ethyl acrylate, methyl methacrylate and trimethylammonioethyl methacrylate). In addition, PEO (MW 2,000,000) was added to the inner layer to enhance the mucoadhesive characteristics. The resulting polymer combination reduced the solubility of the membrane and ensured its structural integrity during hydration, a high degree of swelling (patches taking on 50% of their weight within 15–20 min followed by increases in weight by 70% in 1 h), large surface area, and strong mucoadhesive properties (the in vivo residence time for gingiva and buccal mucosa was 120 min). X-ray diffraction and differential thermal analysis showed that clobetasol was present in an amorphous form in the electrospun patches; therefore, this delivery system has several advantages, including increased solubility, increased dissolution rate, and improved bioavailability, compared to its crystalline equivalent. This patch provided highly localized (systemic absorption was below the level of detection [20 pg/mL] for 6 h) and controlled (20%, 50%, and 80% of the drug was released after 30, 180, and 360 min, respectively) delivery of clobetasol to the mucosal surface. Perez-Gonzalez et al. [22] designed electrospun three-layer patches as a mucoadhesive delivery system for the oromucosal surface to improve the drug safety profile by the unidirectional release of DexP. The resulting system consisted of a drug release layer (DexP+PVP with MW of 40,000), an adhesive layer (PCL+polycarbophil) and a backing layer of PCL (MW 80,000). The developed nanofiber matrices had a porosity of about 60–65% and excellent thermal stability. In vitro tests showed a release of 80% of the loaded DexP in 4 h, and mucoadhesion studies demonstrated excellent mucoadhesion parameters (the detachment force and mucoadhesive strength were 3.5 N and 350 g, respectively). Tonglairoum et al. [47] developed clotrimazole poly-layer patches for improved oral candidiasis application using electrospinning (similar technology can be realized for CS agents). To improve solubility, clotrimazole was loaded into PVP (MW 1,300,000)/HPβCD fibers (inner drug-containing layer). Next, the inner layer was coated with PVA/chitosan (DDA 0.85, MW 110,000) or PVA/cysteine-chitosan on each side to create sandwich nanofibers to increase the mucoadhesion and to achieve a controlled release of the drug from the patch. Scanning electron microscopy showed that the inner fibers had a diameter of 470 nm, and the PVA/chitosan and PVA/cysteine-chitosan coated fibers had a diameter of 190 and 200 nm, respectively. The PVA/cysteine-chitosan coated patches exhibited better mechanical properties (Young’s modulus was about 3 MPa) than the PVA/chitosan coated patches due to the higher flexibility of the PVA/cysteine-chitosan nanofibers compared with the PVA/chitosan nanofibers. In addition, the PVA/cysteine-chitosan coated nanofibers had a higher ex vivo mucoadhesive strength (0.54 g) compared with the PVA/chitosan coated nanofibers (0.43 g) due to the presence of the thiol groups. The designed patches were characterized by a modified release: 40–60% of clotrimazole was released within 1 h, and then sustained release was maintained for 8 h.

### 6.2. 3D Printing Technology 

3D printing by syringe extrusion is currently of great interest for various medical applications, including oral patch development, because this strategy allows the printing of semi-solid formulations (gels and pastes) at room temperature using a wide range of polymers, as well as the loading of different drugs and programming of drug release by varying the matrix geometry and the polymer type and amount [145,146,147,148]. High molecular weight biopolymers, such as chitosan, ALG, hyaluronic acid, gelatin, and collagen, are used as bioinks for 3D printing of mucoadhesive oral patches with controlled drug release due to their printability, biocompatibility, and biodegradability [149,150,151]. 

Nanocellulose can also be used to modify the rheological properties of biopolymers and increase the strength of the resulting materials [152,153].

For example, Olmos-Justea et al. [41] created a 3D-printed patch with a hydrophobic drug (curcumin) based on ALG (MW 240,000) and cellulose nanofibers for local application in the oral cavity. The printed matrices were also sublimated to remove water and form porous aerogels. The 3–5% nanocellulose content provided suitable viscoelastic characteristics for successful printing, thereby increasing shape fidelity and structural integrity, as well as preventing the collapse of printed samples. The obtained systems had high mechanical strength (Young’s modulus of 23–28 MPa, and compressive strength of about 3 MPa) and a high swelling degree of 1000–1200%. An in vitro study (PBS, 37 °C) of drug release kinetics showed that the rate of curcumin release from fabricated freeze-dried printed patches ranged from 100% in 6 h (3–4% nanocellulose) to 50% in 24 h (5% nanocellulose), depending on the degradation rate, porosity, and swelling rate of the matrices.

Bom et al. [42] developed 3D hydrogel patches with improved drug delivery properties based on ALG (medium-viscosity ≥ 2000 cP) and low-viscosity pregelatinized modified starch for topical application. The incorporation of starch into the ALG matrix (30–50% of the ALG mass) led to a reorganization of its structure and to an increase in the porosity (the size and quantity of open pores), thereby preserving the structural stability of the 3D patches. The obtained polymeric systems exhibited a modified release profile of the model drug rhodamine B that included an initial burst effect in the first 30 min and then sustained release for 6 h. The excellent starch swelling properties increased the rate of drug release, as 90% and 70% of rhodamine B was released from ALG patches and ALG-starch patches, respectively, after 6 h. Thus, various innovative technologies make it possible to obtain oromucosal patches with desired properties (Table 5).

## 7. Conclusions and Future Perspectives

This review highlights the importance of using an integrated approach that takes into account all biopharmaceutical factors and the desired technological and pharmacological characteristics of the resulting dosage form when producing polymeric patches for oromucosal delivery of topical CS. The most important property of the drug is its safety and efficacy, and these are provided in oromucosal patches with CS (i) by the development of multilayer systems with a protective layer impermeable to the active pharmaceutical substance and unidirectional release (ii) by biopolymers with improved mucoadhesive properties (iii) by the use of special technological techniques to ensure optimal drug loading, uniform distribution, and subsequent controlled and prolonged drug release.

Promising trends are the modification of natural, non-toxic and biocompatible polymers and the expansion of the line of polymers with enhanced mucoadhesive properties (generation two mucoadhesive polymers) to obtain patches with improved bioadhesion. 

An important approach is to choose a preparation method for polymer patches that achieves optimal physical and chemical characteristics, such as mechanical strength, elasticity, porosity, and swellability. In this respect, both electrospinning and 3D printing technologies are of interest. 

One important condition for successful therapy is patient compliance, and this can be assured by the use of excipients, such as local anesthetics, to control pain and factors that correct the unpleasant taste of the drugs. 

In summary, patches as an oromucosal delivery system have great therapeutic potential, as they represent ideal examples of safe and effective dosage forms that improve local CS therapy.

## Figures and Tables

**Table 1:** Comparative characteristics of polymeric patches and traditional dosage forms.

Polymeric Patches	Traditional Dosage Forms
Enhanced mucoadhesion Extended drug residence time	The rapid removal of the drug from the damaged site Reducing the effectiveness of therapy
Controlled and modified release Rational use of the drug dose	Uncontrolled drug release
Targeted drug release and distribution in the mucosa Decreasing the therapeutic dose	The total dose of the drug enters the oral cavity immediately and is partially misused
Low systemic absorption of the drug Improving the safety profile of the drug	Part of the drug enters the gastrointestinal tract and causes toxic systemic effects
Maximum patient compliance and comfort Pain reduction	The need for frequent use of the drug and the inconvenience to the patient

**Table 2:** Effect of biopharmaceutical factors on the effectiveness of patches as a modified dosage form.

Biopharmaceutical Factor	Area of Influence
Physical state of the drug substance	Release and dissolution rates Degree of permeability
Nature of the excipient	Mechanical properties Mucoadhesion properties Drug residence time in the target site Controlling the drug release
Type of patch (single-layer or poly-layer)	Programming the drug release Directional diffusion of the drug into the damaged site High local bioavailability Low systemic toxicity Improved drug safety profile
Method of patch production	Swelling and porosity Mucoadhesion Drug release profile

**Table 3:** Factors that increase bioadhesion of polymeric patches.

Polymer Characteristic	Influence on Mucoadhesion
Carboxyl and hydroxyl functional groups	Forming hydrogen bonds
Positive surface charge	Ionic interaction
Wettability High viscosity High degree of swelling	Hydrogel-forming properties
Polymer chain length and flexibility	Binding and entanglement to the mucoadhesive reticulum
Degree of cross-linking	Preservation of the polymer structure during swelling Polymer/mucosal interpenetration Controlled drug release
Spatial conformation	Facilitates the interaction of adhesive groups with the substrate

**Table 4:** Approaches to modifying drug release from polymeric patches.

Solubility of CS	Modification Method
Hydrophilic drug	Use of hydrophilic polymer matrices Formation of CD/CS complexes (host/guest interaction)
Hydrophobic drugs	Using nanoparticles or liposomes to load hydrophobic components Forming inclusion complexes based on CD and CD derivatives Dissolving CS in suitable solvents and co-solvents Sonication

**Table 5:** The main advantages of patches obtained by various innovative techniques.

Technique	Advantages
Electrospinning	Creating poly-layer patches Localized drug delivery Unidirectional drug release High local bioavailability Low systemic toxicity High porosity and surface area Enhanced mucoadhesion
3D printing	Use of polymers with high MW Improved bioadhesion Loading both hydrophobic and hydrophilic drugs Programmed drug release

## Data Availability

Not applicable.

## References

[1] Schäfer-Korting M., Kleuser B., Ahmed M., Höltje H.-D., Korting H.C. (2005). Glucocorticoids for human skin: New aspects of the mechanism of action. Ski. Pharmacol. Physiol..

[2] Frangos J.E., Kimball A.B. (2008). Clobetasol propionate emollient formulation foam in the treatment of corticosteroid-responsive dermatoses. Expert Opin. Pharmacother..

[3] Rotaru D., Chisnoiu R., Picos A.M., Picos A., Chisnoiu A. (2020). Treatment trends in oral lichen planus and oral lichenoid lesions. Exp. Ther. Med..

[4] D’Angelo I., Fraix A., Ungaro F., Quaglia F., Miro A. (2017). Poly (ethylene oxide)/hydroxypropyl-β-cyclodextrin films for oromucosal delivery of hydrophilic drugs. Int. J. Pharm..

[5] Gupta S., Ghosh S., Gupta S. (2017). Interventions for the management of oral lichen planus: A review of the conventional and novel therapies. Oral Dis..

[6] Zborowski J., Kida D., Szarwaryn A., Nartowski K., Rak P., Jurczyszyn K., Konopka T. (2021). A comparison of clinical efficiency of photodynamic therapy and topical corticosteroid in treatment of oral lichen planus: A split-mouth randomised controlled study. J. Clin. Med..

[7] Wiedersberg S., Leopold C.S., Guy R.H. (2008). Bioavailability and bioequivalence of topical glucocorticoids. Eur. J. Pharm. Biopharm..

[8] Kwatra G., Mukhopadhyay S. (2018). Topical Corticosteroids: Pharmacology. A Treatise on Topical Corticosteroids in Dermatology.

[9] Bagan J., Compilato D., Paderni C., Campisi G., Panzarella V., Picciotti M., Lorenzini G., Di Fede O. (2012). Topical therapies for oral lichen planus management and their efficacy: A narrative review. Curr. Pharm. Des..

[10] Irene B., Yolanda J., Ariadna C. (2014). Treatment of recurrent aphthous stomatitis. J. Clin. Exp. Dent..

[11] Varoni E.M., Molteni A., Sardella A., Carrassi A., Di Candia D., Gigli F., Lodi F., Lodi G. (2012). Pharmacokinetics study about topical clobetasol on oral mucosa. J. Oral Pathol. Med..

[12] Dubashynskaya N.V., Bokatyi A.N., Skorik Y.A. (2021). Dexamethasone conjugates: Synthetic approaches and medical prospects. Biomedicines.

[13] Dubashynskaya N.V., Bokatyi A.N., Golovkin A.S., Kudryavtsev I.V., Serebryakova M.K., Trulioff A.S., Dubrovskii Y.A., Skorik Y.A. (2021). Synthesis and characterization of novel succinyl chitosan-dexamethasone conjugates for potential intravitreal dexamethasone delivery. Int. J. Mol. Sci..

[14] Dubashynskaya N.V., Golovkin A.S., Kudryavtsev I.V., Prikhodko S.S., Trulioff A.S., Bokatyi A.N., Poshina D.N., Raik S.V., Skorik Y.A. (2020). Mucoadhesive cholesterol-chitosan self-assembled particles for topical ocular delivery of dexamethasone. Int. J. Biol. Macromol..

[15] Decani S., Federighi V., Baruzzi E., Sardella A., Lodi G. (2014). Iatrogenic cushing’s syndrome and topical steroid therapy: Case series and review of the literature. J. Dermatol. Treat..

[16] Glines K.R., Stiff K.M., Freeze M., Cline A., Strowd L.C., Feldman S.R. (2019). An update on the topical and oral therapy options for treating pediatric atopic dermatitis. Expert Opin. Pharmacother..

[17] Holpuch A.S., Hummel G.J., Tong M., Seghi G.A., Pei P., Ma P., Mumper R.J., Mallery S.R. (2010). Nanoparticles for local drug delivery to the oral mucosa: Proof of principle studies. Pharm. Res..

[18] Campos J.C., Ferreira D.C., Lima S., Reis S., Costa P.J. (2019). Swellable polymeric particles for the local delivery of budesonide in oral mucositis. Int. J. Pharm..

[19] Dukovski B.J., Plantić I., Čunčić I., Krtalić I., Juretić M., Pepić I., Lovrić J., Hafner A. (2017). Lipid/alginate nanoparticle-loaded in situ gelling system tailored for dexamethasone nasal delivery. Int. J. Pharm..

[20] Siddique M.I., Katas H., Amin M.C.I.M., Ng S.-F., Zulfakar M.H., Buang F., Jamil A. (2015). Minimization of local and systemic adverse effects of topical glucocorticoids by nanoencapsulation: In vivo safety of hydrocortisone–hydroxytyrosol loaded chitosan nanoparticles. J. Pharm. Sci..

[21] Rohani Shirvan A., Hemmatinejad N., Bahrami S.H., Bashari A. (2021). Fabrication of multifunctional mucoadhesive buccal patch for drug delivery applications. J. Biomed. Mater. Res. Part A.

[22] Pérez-González G.L., Villarreal-Gómez L.J., Olivas-Sarabia A., Valdez R., Cornejo-Bravo J.M. (2021). Development, characterization, and in vitro assessment of multilayer mucoadhesive system containing dexamethasone sodium phosphate. Int. J. Polym. Mater. Polym. Biomater..

[23] Paderni C., Compilato D., Giannola L.I., Campisi G. (2012). Oral local drug delivery and new perspectives in oral drug formulation. Oral Surg. Oral Med. Oral Pathol. Oral Radiol..

[24] Pérez-González G.L., Villarreal-Gómez L.J., Serrano-Medina A., Torres-Martínez E.J., Cornejo-Bravo J.M. (2019). Mucoadhesive electrospun nanofibers for drug delivery systems: Applications of polymers and the parameters’ roles. Int. J. Nanomed..

[25] Vigani B., Rossi S., Sandri G., Bonferoni M.C., Caramella C.M., Ferrari F. (2020). Recent advances in the development of in situ gelling drug delivery systems for non-parenteral administration routes. Pharmaceutics.

[26] Lindert S., Breitkreutz J. (2017). Oromucosal multilayer films for tailor-made, controlled drug delivery. Expert Opin. Drug Deliv..

[27] Salamat-Miller N., Chittchang M., Johnston T.P. (2005). The use of mucoadhesive polymers in buccal drug delivery. Adv. Drug Deliv. Rev..

[28] Lee J.W., Park J.H., Robinson J.R. (2000). Bioadhesive-based dosage forms: The next generation. J. Pharm. Sci..

[29] Morales J.O., McConville J.T. (2011). Manufacture and characterization of mucoadhesive buccal films. Eur. J. Pharm. Biopharm..

[30] Borges A.F., Silva C., Coelho J.F., Simões S. (2015). Oral films: Current status and future perspectives: I—Galenical development and quality attributes. J. Control. Release.

[31] Colley H., Said Z., Santocildes-Romero M., Baker S., D’Apice K., Hansen J., Madsen L.S., Thornhill M., Hatton P., Murdoch C. (2018). Pre-clinical evaluation of novel mucoadhesive bilayer patches for local delivery of clobetasol-17-propionate to the oral mucosa. Biomaterials.

[32] Edmans J.G., Clitherow K.H., Murdoch C., Hatton P.V., Spain S.G., Colley H.E. (2020). Mucoadhesive electrospun fibre-based technologies for oral medicine. Pharmaceutics.

[33] Hosseinpour-Moghadam R., Mehryab F., Torshabi M., Haeri A. (2021). Applications of novel and nanostructured drug delivery systems for the treatment of oral cavity diseases. Clin. Ther..

[34] Owji N., Mandakhbayar N., Gregory D.A., Marcello E., Kim H.-w., Roy I., Knowles J.C. (2021). Mussel inspired chemistry and bacteria derived polymers for oral mucosal adhesion and drug delivery. Front. Bioeng. Biotechnol..

[35] Csóka I., Pallagi E., Paál T.L. (2018). Extension of quality-by-design concept to the early development phase of pharmaceutical r&d processes. Drug Discov. Today.

[36] Mašková E., Kubová K., Raimi-Abraham B.T., Vllasaliu D., Vohlídalová E., Turánek J., Mašek J. (2020). Hypromellose–a traditional pharmaceutical excipient with modern applications in oral and oromucosal drug delivery. J. Control. Release.

[37] Laffleur F., Krouská J., Tkacz J., Pekař M., Aghai F., Netsomboon K. (2018). Buccal adhesive films with moisturizer-the next level for dry mouth syndrome?. Int. J. Pharm..

[38] Santocildes-Romero M.E., Hadley L., Clitherow K.H., Hansen J., Murdoch C., Colley H.E., Thornhill M.H., Hatton P.V. (2017). Fabrication of electrospun mucoadhesive membranes for therapeutic applications in oral medicine. ACS Appl. Mater. Interfaces.

[39] Okeke O.C., Boateng J.S. (2016). Composite hpmc and sodium alginate based buccal formulations for nicotine replacement therapy. Int. J. Biol. Macromol..

[40] Bandi S.P., Venuganti V.V.K. (2021). Functionalized polymeric patch for localized oxaliplatin delivery to treat gastric cancer. Mater. Sci. Eng. C.

[41] Olmos-Juste R., Alonso-Lerma B., Pérez-Jiménez R., Gabilondo N., Eceiza A. (2021). 3d printed alginate-cellulose nanofibers based patches for local curcumin administration. Carbohydr. Polym..

[42] Bom S., Santos C., Barros R., Martins A.M., Paradiso P., Cláudio R., Pinto P.C., Ribeiro H.M., Marto J. (2020). Effects of starch incorporation on the physicochemical properties and release kinetics of alginate-based 3d hydrogel patches for topical delivery. Pharmaceutics.

[43] Paris A.-L., Caridade S., Colomb E., Bellina M., Boucard E., Verrier B., Monge C. (2021). Sublingual protein delivery by a mucoadhesive patch made of natural polymers. Acta Biomater..

[44] Zhou A., Zhang Y., Zhang X., Deng Y., Huang D., Huang C., Qu Q. (2022). Quaternized chitin/tannic acid bilayers layer-by-layer deposited poly (lactic acid)/polyurethane nanofibrous mats decorated with photoresponsive complex and silver nanoparticles for antibacterial activity. Int. J. Biol. Macromol..

[45] Smart J.D. (2005). The basics and underlying mechanisms of mucoadhesion. Adv. Drug Deliv. Rev..

[46] Nair A.B., Kumria R., Harsha S., Attimarad M., Al-Dhubiab B.E., Alhaider I.A. (2013). In vitro techniques to evaluate buccal films. J. Control. Release.

[47] Tonglairoum P., Ngawhirunpat T., Rojanarata T., Panomsuk S., Kaomongkolgit R., Opanasopit P. (2015). Fabrication of mucoadhesive chitosan coated polyvinylpyrrolidone/cyclodextrin/clotrimazole sandwich patches for oral candidiasis. Carbohydr. Polym..

[48] Khan G., Yadav S.K., Patel R.R., Nath G., Bansal M., Mishra B. (2016). Development and evaluation of biodegradable chitosan films of metronidazole and levofloxacin for the management of periodontitis. Aaps Pharmscitech.

[49] Schattling P., Taipaleenmäki E., Zhang Y., Städler B. (2017). A polymer chemistry point of view on mucoadhesion and mucopenetration. Macromol. Biosci..

[50] Wang L., Zhou Y., Wu M., Wu M., Li X., Gong X., Chang J., Zhang X. (2018). Functional nanocarrier for drug and gene delivery via local administration in mucosal tissues. Nanomedicine.

[51] Şenel S. (2021). An overview of physical, microbiological and immune barriers of oral mucosa. Int. J. Mol. Sci..

[52] Ibrahim N.A., Elmorshedy K.E., Radwan D.A., Buabeid M.A. (2022). The impact of oral ciprofloxacin on the structure and functions of rat gastric mucosa. Saudi J. Biol. Sci..

[53] Lin D., Yang L., Wen L., Lu H., Chen Q., Wang Z. (2021). Crosstalk between the oral microbiota, mucosal immunity, and the epithelial barrier regulates oral mucosal disease pathogenesis. Mucosal. Immunol..

[54] Cruchley A.T., Bergmeier L.A., Bergmeier L.A. (2018). Structure and Functions of the Oral Mucosa. Oral Mucosa in Health and Disease: A Concise Handbook.

[55] Cone R.A. (2009). Barrier properties of mucus. Adv. Drug Deliv. Rev..

[56] Bruschi M.L., de Souza Ferreira S.B., da Silva J.B. (2020). Mucoadhesive and Mucus-Penetrating Polymers for Drug Delivery. Nanotechnology for Oral Drug Delivery.

[57] Brown T.D., Whitehead K.A., Mitragotri S. (2020). Materials for oral delivery of proteins and peptides. Nat. Rev. Mater..

[58] Pandey M., Choudhury H., Ying J.N.S., Ling J.F.S., Ting J., Ting J.S.S., Zhia Hwen I.K., Suen H.W., Samsul Kamar H.S., Gorain B. (2022). Mucoadhesive nanocarriers as a promising strategy to enhance intracellular delivery against oral cavity carcinoma. Pharmaceutics.

[59] Brannigan R.P., Khutoryanskiy V.V. (2019). Progress and current trends in the synthesis of novel polymers with enhanced mucoadhesive properties. Macromol. Biosci..

[60] Huang Y., Leobandung W., Foss A., Peppas N.A. (2000). Molecular aspects of muco-and bioadhesion:: Tethered structures and site-specific surfaces. J. Control. Release.

[61] Mansuri S., Kesharwani P., Jain K., Tekade R.K., Jain N. (2016). Mucoadhesion: A promising approach in drug delivery system. React. Funct. Polym..

[62] Do Nascimento E.G., de Azevedo E.P., Alves-Silva M.F., Aragão C.F.S., Fernandes-Pedrosa M.F., da Silva-Junior A.A. (2020). Supramolecular aggregates of cyclodextrins with co-solvent modulate drug dispersion and release behavior of poorly soluble corticosteroid from chitosan membranes. Carbohydr. Polym..

[63] Ghalayani Esfahani A., Altomare L., Varoni E.M., Bertoldi S., Farè S., De Nardo L. (2019). Electrophoretic bottom up design of chitosan patches for topical drug delivery. J. Mater. Sci. Mater. Med..

[64] Ways T.M.M., Lau W.M., Khutoryanskiy V.V. (2018). Chitosan and its derivatives for application in mucoadhesive drug delivery systems. Polymers.

[65] Kolawole O.M., Lau W.M., Khutoryanskiy V.V. (2018). Methacrylated chitosan as a polymer with enhanced mucoadhesive properties for transmucosal drug delivery. Int. J. Pharm..

[66] Kumar K., Dhawan N., Sharma H., Vaidya S., Vaidya B. (2014). Bioadhesive polymers: Novel tool for drug delivery. Artif. Cells Nanomed. Biotechnol..

[67] Jones D.S., Bruschi M.L., de Freitas O., Gremião M.P.D., Lara E.H.G., Andrews G.P. (2009). Rheological, mechanical and mucoadhesive properties of thermoresponsive, bioadhesive binary mixtures composed of poloxamer 407 and carbopol 974p designed as platforms for implantable drug delivery systems for use in the oral cavity. Int. J. Pharm..

[68] Ramineni S.K., Cunningham L.L., Dziubla T.D., Puleo D.A. (2013). Competing properties of mucoadhesive films designed for localized delivery of imiquimod. Biomater. Sci..

[69] Antosik A.K., Miądlicki P., Wilpiszewska K., Markowska-Szczupak A., Koren Z.C., Wróblewska A. (2021). Polysaccharide films modified by compounds of natural origin and silver having potential medical applications. Cellulose.

[70] Kiroshka V.V., Petrova V.A., Chernyakov D.D., Bozhkova Y.O., Kiroshka K.V., Baklagina Y.G., Romanov D.P., Kremnev R.V., Skorik Y.A. (2017). Influence of chitosan-chitin nanofiber composites on cytoskeleton structure and the proliferation of rat bone marrow stromal cells. J. Mater. Sci. Mater. Med..

[71] Petrova V.A., Chernyakov D.D., Poshina D.N., Gofman I.V., Romanov D.P., Mishanin A.I., Golovkin A.S., Skorik Y.A. (2019). Electrospun bilayer chitosan/hyaluronan material and its compatibility with mesenchymal stem cells. Materials.

[72] Zienkiewicz-Strzałka M., Deryło-Marczewska A., Skorik Y.A., Petrova V.A., Choma A., Komaniecka I. (2019). Silver nanoparticles on chitosan/silica nanofibers: Characterization and antibacterial activity. Int. J. Mol. Sci..

[73] Petrova V.A., Golovkin A.S., Mishanin A.I., Romanov D.P., Chernyakov D.D., Poshina D.N., Skorik Y.A. (2020). Cytocompatibility of bilayer scaffolds electrospun from chitosan/alginate-chitin nanowhiskers. Biomedicines.

[74] Safdar R., Omar A.A., Arunagiri A., Regupathi I., Thanabalan M. (2019). Potential of chitosan and its derivatives for controlled drug release applications–a review. J. Drug Deliv. Sci. Technol..

[75] Lopes S.A., Veiga I.G., Bierhalz A.C.K., Pires A.L.R., Moraes Â.M. (2019). Physicochemical properties and release behavior of indomethacin-loaded polysaccharide membranes. Int. J. Polym. Mater. Polym. Biomater..

[76] Qu R., Zhang W., Liu N., Zhang Q., Liu Y., Li X., Wei Y., Feng L. (2018). Antioil ag3po4 nanoparticle/polydopamine/al2o3 sandwich structure for complex wastewater treatment: Dynamic catalysis under natural light. ACS Sustain. Chem. Eng..

[77] Sahariah P., Másson M. (2017). Antimicrobial chitosan and chitosan derivatives: A review of the structure–activity relationship. Biomacromolecules.

[78] Guo Z., Xing R., Liu S., Zhong Z., Ji X., Wang L., Li P. (2008). The influence of molecular weight of quaternized chitosan on antifungal activity. Carbohydr. Polym..

[79] Kumar A., Vimal A., Kumar A. (2016). Why chitosan? From properties to perspective of mucosal drug delivery. Int. J. Biol. Macromol..

[80] Sogias I.A., Williams A.C., Khutoryanskiy V.V. (2008). Why is chitosan mucoadhesive?. Biomacromolecules.

[81] Xing K., Xing Y., Liu Y., Zhang Y., Shen X., Li X., Miao X., Feng Z., Peng X., Qin S. (2018). Fungicidal effect of chitosan via inducing membrane disturbance against ceratocystis fimbriata. Carbohydr. Polym..

[82] Yin M., Wang Y., Zhang Y., Ren X., Qiu Y., Huang T.-S. (2020). Novel quaternarized n-halamine chitosan and polyvinyl alcohol nanofibrous membranes as hemostatic materials with excellent antibacterial properties. Carbohydr. Polym..

[83] Meng D., Garba B., Ren Y., Yao M., Xia X., Li M., Wang Y. (2020). Antifungal activity of chitosan against aspergillus ochraceus and its possible mechanisms of action. Int. J. Biol. Macromol..

[84] Ma Q., Zhang Y., Critzer F., Davidson P.M., Zivanovic S., Zhong Q. (2016). Physical, mechanical, and antimicrobial properties of chitosan films with microemulsions of cinnamon bark oil and soybean oil. Food Hydrocoll..

[85] Palma S.D., Tartara L.I., Quinteros D., Allemandi D.A., Longhi M.R., Granero G.E. (2009). An efficient ternary complex of acetazolamide with hp-ß-cd and tea for topical ocular administration. J. Control. Release.

[86] Abramov E., Schwob O., Benny O. (2019). Film-and ointment-based delivery systems for the transdermal delivery of tnp-470. Polym. Adv. Technol..

[87] Laredo J.-D., Mosseri J., Nizard R. (2017). Percutaneous nailing and cementoplasty for palliative management of supra-acetabular iliac wing metastases: A case report. JBJS Case Connect..

[88] De Medeiros A.S., Zoppi A., Barbosa E.G., Oliveira J.I., Fernandes-Pedrosa M.F., Longhi M.R., da Silva-Júnior A.A. (2016). Supramolecular aggregates of oligosaccharides with co-solvents in ternary systems for the solubilizing approach of triamcinolone. Carbohydr. Polym..

[89] George D., Maheswari P.U., Begum K.M.S. (2020). Chitosan-cellulose hydrogel conjugated with l-histidine and zinc oxide nanoparticles for sustained drug delivery: Kinetics and in-vitro biological studies. Carbohydr. Polym..

[90] Cazorla-Luna R., Martín-Illana A., Notario-Pérez F., Ruiz-Caro R., Veiga M.-D. (2021). Naturally occurring polyelectrolytes and their use for the development of complex-based mucoadhesive drug delivery systems: An overview. Polymers.

[91] Dodero A., Alloisio M., Castellano M., Vicini S. (2020). Multilayer alginate–polycaprolactone electrospun membranes as skin wound patches with drug delivery abilities. ACS Appl. Mater. Interfaces.

[92] Marioane C.-A., Bunoiu M., Mateescu M., Sfîrloagă P., Vlase G., Vlase T. (2021). Preliminary study for the preparation of transmucosal or transdermal patches with acyclovir and lidocaine. Polymers.

[93] Szekalska M., Puciłowska A., Szymańska E., Ciosek P., Winnicka K. (2016). Alginate: Current use and future perspectives in pharmaceutical and biomedical applications. Int. J. Polym. Sci..

[94] Chinwala M.G., Lin S. (2010). Application of hydrogel polymers for development of thyrotropin releasing hormone-loaded adhesive buccal patches. Pharm. Dev. Technol..

[95] Szabó B., Sebe I., Kállai N., Süvegh K., Zelkó R. (2013). Comparison of the micro-and macrostructural characteristics of biopolymer cast films. Eur. Polym. J..

[96] Javanbakht S., Shaabani A. (2019). Carboxymethyl cellulose-based oral delivery systems. Int. J. Biol. Macromol..

[97] Tedesco M.P., dos Santos Garcia V.A., Borges J.G., Osiro D., Vanin F.M., Yoshida C.M.P., de Carvalho R.A. (2021). Production of oral films based on pre-gelatinized starch, cmc and hpmc for delivery of bioactive compounds extract from acerola industrial waste. Ind. Crops Prod..

[98] Pettignano A., Charlot A., Fleury E. (2019). Carboxyl-functionalized derivatives of carboxymethyl cellulose: Towards advanced biomedical applications. Polym. Rev..

[99] Ramineni S.K., Cunningham L.L., Dziubla T.D., Puleo D.A. (2013). Development of imiquimod-loaded mucoadhesive films for oral dysplasia. J. Pharm. Sci..

[100] Göbel A., da Silva J.B., Cook M., Breitkreutz J. (2021). Development of buccal film formulations and their mucoadhesive performance in biomimetic models. Int. J. Pharm..

[101] Lam H.T., Zupančič O., Laffleur F., Bernkop-Schnürch A. (2021). Mucoadhesive properties of polyacrylates: Structure–function relationship. Int. J. Adhes. Adhes..

[102] Smart J.D. (2004). Recent developments in the use of bioadhesive systems for delivery of drugs to the oral cavity. Crit. Rev. ™ Ther. Drug Carr. Syst..

[103] Woertz C., Preis M., Breitkreutz J., Kleinebudde P. (2013). Assessment of test methods evaluating mucoadhesive polymers and dosage forms: An overview. Eur. J. Pharm. Biopharm..

[104] Leitner V., Marschütz M., Bernkop-Schnürch A. (2003). Mucoadhesive and cohesive properties of poly (acrylic acid)-cysteine conjugates with regard to their molecular mass. Eur. J. Pharm. Sci..

[105] Hägerström H., Edsman K. (2001). Interpretation of mucoadhesive properties of polymer gel preparations using a tensile strength method. J. Pharm. Pharmacol..

[106] Netsomboon K., Jalil A., Laffleur F., Hupfauf A., Gust R., Bernkop-Schnürch A. (2020). Thiolated chitosans: Are cys-cys ligands key to the next generation?. Carbohydr. Polym..

[107] Puri V., Sharma A., Kumar P., Singh I. (2020). Thiolation of biopolymers for developing drug delivery systems with enhanced mechanical and mucoadhesive properties: A review. Polymers.

[108] Laffleur F., Bernkop-Schnürch A. (2018). Evaluation of dermal adhesive formulations for topical application. Eur. J. Pharm. Biopharm..

[109] Grießinger J.A., Bonengel S., Partenhauser A., Ijaz M., Bernkop-Schnürch A. (2017). Thiolated polymers: Evaluation of their potential as dermoadhesive excipients. Drug Dev. Ind. Pharm..

[110] Jelkmann M., Bonengel S., Menzel C., Markovic S., Bernkop-Schnürch A. (2018). New perspectives of starch: Synthesis and in vitro assessment of novel thiolated mucoadhesive derivatives. Int. J. Pharm..

[111] Griesser J., Hetényi G., Bernkop-Schnürch A. (2018). Thiolated hyaluronic acid as versatile mucoadhesive polymer: From the chemistry behind to product developments—What are the capabilities?. Polymers.

[112] Knoll P., Le N.-M.N., Wibel R., Baus R.A., Kali G., Asim M.H., Bernkop-Schnürch A. (2021). Thiolated pectins: In vitro and ex vivo evaluation of three generations of thiomers. Acta Biomater..

[113] Duggan S., O’Donovan O., Owens E., Duggan E., Hughes H., Cummins W. (2016). Comparison of the mucoadhesive properties of thiolated polyacrylic acid to thiolated polyallylamine. Int. J. Pharm..

[114] Özkahraman B., Özbaş Z., Yaşayan G., Akgüner Z.P., Yarımcan F., Alarçin E., Bal-Öztürk A. (2022). Development of mucoadhesive modified kappa-carrageenan/pectin patches for controlled delivery of drug in the buccal cavity. J. Biomed. Mater. Res. Part B Appl. Biomater..

[115] Naz K., Shahnaz G., Ahmed N., Qureshi N.A., Sarwar H.S., Imran M., Khan G.M. (2017). Formulation and in vitro characterization of thiolated buccoadhesive film of fluconazole. Aaps Pharmscitech..

[116] Hanif M., Zaman M. (2017). Thiolation of arabinoxylan and its application in the fabrication of controlled release mucoadhesive oral films. DARU J. Pharm. Sci..

[117] Sonvico F., Clementino A., Buttini F., Colombo G., Pescina S., Stanisçuaski Guterres S., Raffin Pohlmann A., Nicoli S. (2018). Surface-modified nanocarriers for nose-to-brain delivery: From bioadhesion to targeting. Pharmaceutics.

[118] Numata K., Baker P.J. (2014). Synthesis of adhesive peptides similar to those found in blue mussel (*Mytilus edulis*) using papain and tyrosinase. Biomacromolecules.

[119] Ahn B.K. (2017). Perspectives on mussel-inspired wet adhesion. J. Am. Chem. Soc..

[120] Lee H., Dellatore S., Miller W., Messersmith P. (2007). Mussel-inspired surface chemistry for multifunctional coatings haeshin. Science.

[121] Yang Y., Qi P., Ding Y., Maitz M.F., Yang Z., Tu Q., Xiong K., Leng Y., Huang N. (2015). A biocompatible and functional adhesive amine-rich coating based on dopamine polymerization. J. Mater. Chem. B.

[122] Saiz-Poseu J., Mancebo-Aracil J., Nador F., Busqué F., Ruiz-Molina D. (2019). The chemistry behind catechol-based adhesion. Angew. Chem. Int. Ed..

[123] Zhao F., He F., Liu X., Shi J., Liang J., Wang S., Yang C., Liu R. (2020). Metabolic engineering of pseudomonas mendocina nk-01 for enhanced production of medium-chain-length polyhydroxyalkanoates with enriched content of the dominant monomer. Int. J. Biol. Macromol..

[124] Basnett P., Lukasiewicz B., Marcello E., Gura H.K., Knowles J.C., Roy I. (2017). Production of a novel medium chain length poly (3-hydroxyalkanoate) using unprocessed biodiesel waste and its evaluation as a tissue engineering scaffold. Microb. Biotechnol..

[125] Shahid S., Razzaq S., Farooq R. (2021). Polyhydroxyalkanoates: Next generation natural biomolecules and a solution for the world’s future economy. Int. J. Biol. Macromol..

[126] Elmowafy E., Abdal-Hay A., Skouras A., Tiboni M., Casettari L., Guarino V. (2019). Polyhydroxyalkanoate (pha): Applications in drug delivery and tissue engineering. Expert Rev. Med. Devices.

[127] Wang D., Jia M., Wang L., Song S., Feng J., Zhang X. (2017). Chitosan and β-cyclodextrin-epichlorohydrin polymer composite film as a plant healthcare material for carbendazim-controlled release to protect rape against sclerotinia sclerotiorum (lib.) de bary. Materials.

[128] Batista P., Castro P., Madureira A.R., Sarmento B., Pintado M. (2019). Development and characterization of chitosan microparticles-in-films for buccal delivery of bioactive peptides. Pharmaceuticals.

[129] Kutyła M.J., Boehm M.W., Stokes J.R., Shaw P.N., Davies N.M., McGeary R.P., Tuke J., Ross B.P. (2013). Cyclodextrin-crosslinked poly (acrylic acid): Adhesion and controlled release of diflunisal and fluconazole from solid dosage forms. Aaps Pharmscitech..

[130] Miro A., d’Angelo I., Nappi A., La Manna P., Biondi M., Mayol L., Musto P., Russo R., La Rotonda M.I., Ungaro F. (2013). Engineering poly (ethylene oxide) buccal films with cyclodextrin: A novel role for an old excipient?. Int. J. Pharm..

[131] Miro A., Ungaro F., Balzano F., Masi S., Musto P., Lamanna P., Uccello Barretta G., Quaglia F. (2012). Triamcinolone solubilization by (2-hydroxypropyl)-ß-cyclodextrin: A spectroscopic and computational approach. Carbohydr. Polym..

[132] Hafsa J., ali Smach M., Khedher M.R.B., Charfeddine B., Limem K., Majdoub H., Rouatbi S. (2016). Physical, antioxidant and antimicrobial properties of chitosan films containing eucalyptus globulus essential oil. LWT-Food Sci. Technol..

[133] Chonkar A.D., Rao J.V., Managuli R.S., Mutalik S., Dengale S., Jain P., Udupa N. (2016). Development of fast dissolving oral films containing lercanidipine hcl nanoparticles in semicrystalline polymeric matrix for enhanced dissolution and ex vivo permeation. Eur. J. Pharm. Biopharm..

[134] Haghju S., Beigzadeh S., Almasi H., Hamishehkar H. (2016). Chitosan films incorporated with nettle (urtica dioica l.) extract-loaded nanoliposomes: I. Physicochemical characterisation and antimicrobial properties. J. Microencapsul..

[135] Medina E., Caro N., Abugoch L., Gamboa A., Díaz-Dosque M., Tapia C. (2019). Chitosan thymol nanoparticles improve the antimicrobial effect and the water vapour barrier of chitosan-quinoa protein films. J. Food Eng..

[136] Jug M., Kosalec I., Maestrelli F., Mura P. (2012). Development of low methoxy amidated pectin-based mucoadhesive patches for buccal delivery of triclosan: Effect of cyclodextrin complexation. Carbohydr. Polym..

[137] Kalaycıoğlu Z., Torlak E., Akın-Evingür G., Özen İ., Erim F.B. (2017). Antimicrobial and physical properties of chitosan films incorporated with turmeric extract. Int. J. Biol. Macromol..

[138] Priyadarshi R., Kumar B., Negi Y.S. (2018). Chitosan film incorporated with citric acid and glycerol as an active packaging material for extension of green chilli shelf life. Carbohydr. Polym..

[139] Tomić K., Veeman W.S., Boerakker M., Litvinov V.M., Dias A.A. (2008). Lateral and rotational mobility of some drug molecules in a poly (ethylene glycol) diacrylate hydrogel and the effect of drug-cyclodextrin complexation. J. Pharm. Sci..

[140] Bibby D.C., Davies N.M., Tucker I.G. (2000). Mechanisms by which cyclodextrins modify drug release from polymeric drug delivery systems. Int. J. Pharm..

[141] Potaś J., Szymańska E., Wróblewska M., Kurowska I., Maciejczyk M., Basa A., Wolska E., Wilczewska A.Z., Winnicka K. (2021). Multilayer films based on chitosan/pectin polyelectrolyte complexes as novel platforms for buccal administration of clotrimazole. Pharmaceutics.

[142] Alhijjaj M., Bouman J., Wellner N., Belton P., Qi S. (2015). Creating drug solubilization compartments via phase separation in multicomponent buccal patches prepared by direct hot melt extrusion–injection molding. Mol. Pharm..

[143] Ahmadi P., Jahanban-Esfahlan A., Ahmadi A., Tabibiazar M., Mohammadifar M. (2022). Development of ethyl cellulose-based formulations: A perspective on the novel technical methods. Food Rev. Int..

[144] Dott C., Tyagi C., Tomar L.K., Choonara Y.E., Kumar P., du Toit L.C., Pillay V. (2013). A mucoadhesive electrospun nanofibrous matrix for rapid oramucosal drug delivery. J. Nanomater..

[145] Placone J.K., Engler A.J. (2018). Recent advances in extrusion-based 3d printing for biomedical applications. Adv. Healthc. Mater..

[146] Azad M.A., Olawuni D., Kimbell G., Badruddoza A.Z.M., Hossain M.S., Sultana T. (2020). Polymers for extrusion-based 3d printing of pharmaceuticals: A holistic materials–process perspective. Pharmaceutics.

[147] Goyanes A., Det-Amornrat U., Wang J., Basit A.W., Gaisford S. (2016). 3d scanning and 3d printing as innovative technologies for fabricating personalized topical drug delivery systems. J. Control. Release.

[148] Karavasili C., Eleftheriadis G.K., Gioumouxouzis C., Andriotis E.G., Fatouros D.G. (2021). Mucosal drug delivery and 3d printing technologies: A focus on special patient populations. Adv. Drug Deliv. Rev..

[149] Jammalamadaka U., Tappa K. (2018). Recent advances in biomaterials for 3d printing and tissue engineering. J. Funct. Biomater..

[150] Schwab A., Levato R., D’Este M., Piluso S., Eglin D., Malda J. (2020). Printability and shape fidelity of bioinks in 3d bioprinting. Chem. Rev..

[151] Aguilar-de-Leyva Á., Linares V., Casas M., Caraballo I. (2020). 3d printed drug delivery systems based on natural products. Pharmaceutics.

[152] Heggset E.B., Strand B.L., Sundby K.W., Simon S., Chinga-Carrasco G., Syverud K. (2019). Viscoelastic properties of nanocellulose based inks for 3d printing and mechanical properties of cnf/alginate biocomposite gels. Cellulose.

[153] Siqueira G., Kokkinis D., Libanori R., Hausmann M.K., Gladman A.S., Neels A., Tingaut P., Zimmermann T., Lewis J.A., Studart A.R. (2017). Cellulose nanocrystal inks for 3d printing of textured cellular architectures. Adv. Funct. Mater..

